# Giant cell arteritis presenting as bilateral anterior ischemic optic neuropathy: a biopsy-proven case report in Chinese patient

**DOI:** 10.1186/s12886-018-0953-5

**Published:** 2018-10-30

**Authors:** Guohong Tian, Weimin Chen, Qian Chen, Min Wang, Guixian Zhao, Zhenxin Li, Jiong Zhang

**Affiliations:** 1 Department of Ophthalmology, Eye Ear Nose and Throat Hospital, Fudan University, Shanghai, 200031 China; 2 Department of Neurology, Shanghai Deji Hospital, Shanghai, 200010 China; 3 Department of Neurology, Huashan Hospital, Fudan University, Shanghai, 200040 China; 4 Department of Rheumatology, Huashan Hospital, Fudan University, Shanghai, 200040 China; 5 NHC Key Laboratory of Myopia, Fudan University, 83 Fenyang Road, Shanghai, 200031 China

**Keywords:** Giant cell arteritis, Anterior ischemic optic neuropathy, Temporal biopsy, Ultrasonography, Asian

## Abstract

**Background:**

Giant cell arteritis (GCA) is a systemic vasculitis of medium and large-size vessels and can led to permanent visual loss in elderly patients. GCA is very rare among Asians. We report a Chinese patient presenting with acute bilateral anterior ischemic optic neuropathy, and the temporal artery biopsy proved the diagnose of GCA.

**Case presentation:**

A 77-year-old Chinese man presented with sudden bilateral blindness for 5 days with a severe headache. Funduscopic examination revealed bilateral optic disc swollen with “chalky white” pallid appearance. The blood tests showed the erythrocyte sedimentation rate (ESR) and C-reactive protein (CRP) elevated dramatically. The color duplex ultrasonography (CDUS) of the superficial temporal artery revealed the inflammation of the vessel wall as a “halo sign”. The temporal artery biopsy was perfumed and the pathology revealed luminal occlusion with multinuclear giant cell infiltration. The patient was treated with intravenous methylprednisolone for 3 days and oral prednisone weaning for 12 months. The visual acuity remained no light perception at one year follow-up.

**Conclusions:**

Although very rare in Asian, GCA can led to permanent blindness in elderly Chinese caused by anterior ischemic optic neuropathy. The noninvasive CDUS might be a promising technique for diagnose GCA in highly suspected patients.

## Background

Giant cell arteritis as a common systemic vasculitis in Western adults, was seen 20 times less frequently in Asian than in Caucasian patients [1–3]. The epidemiological studies have rarely been addressed and according to Kobayashi et al., the prevalence in Japan is 1.47 per 100 000 [4]. Two large sample studies in China mainland showed the clinical manifestations of GCA in Chinese patients were similar to those previously described in literature [5, 6]. Arteritic anterior ischemic optic neuropathy (A-AION) due to GCA, which accounting for 85% of cases of permanent vision loss [7], had not been reported in China so far, except for several presumed cases without biopsy published in non-English literature. We reported an Asian elderly patient with sudden bilateral blindness as initial symptom presented to ophthalmologist, and the afterwards biopsy convinced the pathogenesis of GCA.

## Case presentation

**Fig. 1:**
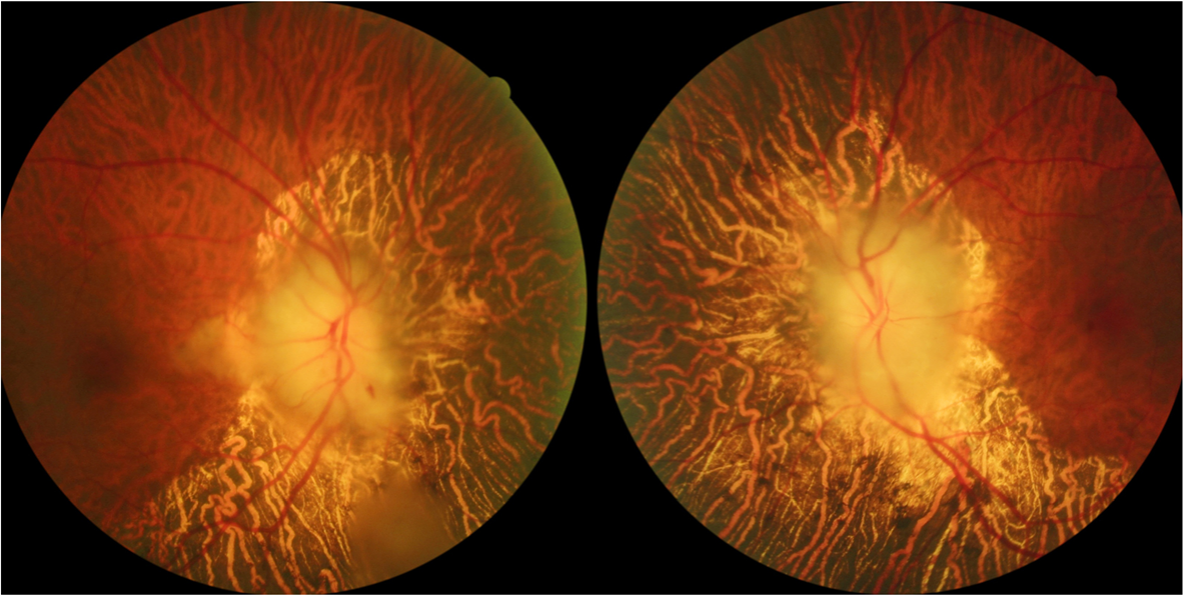
Fundus photographs at presentation of the patient showing severe bilateral optic disc swollen, with “chalky white” pallid appearance; there are splinter hemorrhage and cotton wool spots on the retina in the right eye. The choroid showing diffused atrophy around the optic disc

A 77-year-old Chinese man complained of bilateral, simultaneous onset vision loss for 5 days, accompanied by severe headache on right side and jaw pain. He was a considerable healthy man in the past. The Neuro-Ophthalmological examination revealed the patient to be alert and oriented. The visual acuities were no light perception in both eyes. The pupils were dilated with no reaction to light. Slit lamp examination showed punctate cataract and funduscopic examination revealed diffused swollen of the optic disc bilateral, with pallid “chalky white” appearance; the choroids showing diffused atrophy around the optic disc bilateral (Fig. 1). The extra-ocular motility was full of both eyes. The bilateral superficial temporal arteries were palpable and tenderness.

**Fig. 2:**
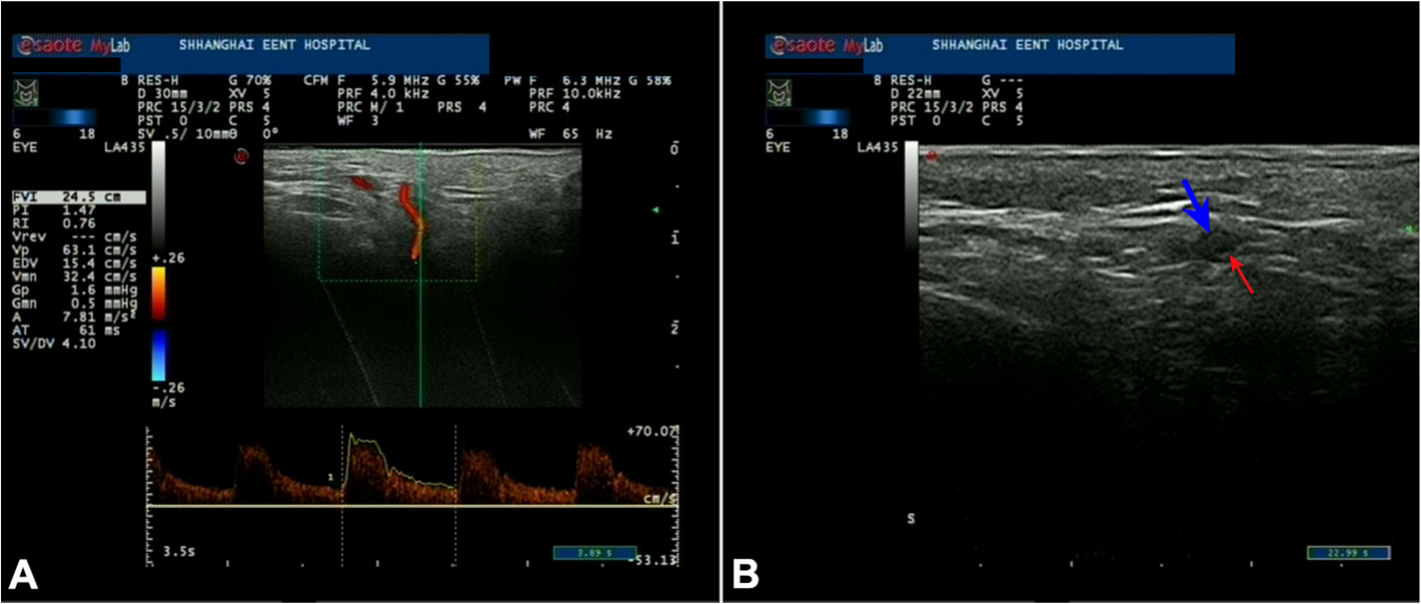
The color duplex ultrasonography showing the right superficial temporal artery of the patient: (**a**): the accelerating of the blood flow indicate the narrowing of the vessel; (**b**): the inflammation of the vessel wall as a hypo-echoic concentric ring, which referred as the “halo sign”. The blue arrow indicates the thickening vessel wall; the red arrow indicates the narrowed luminal

**Fig. 3:**
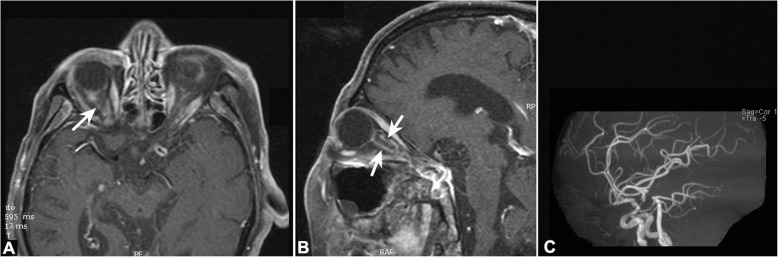
The orbital fat-suppression T1-weighted magnetic resonance imaging with contract showing the enhancement of the optic nerve sheath in the right eye, (**a**):axial and (**b**): sagittal. The white arrows indicate the optic nerve sheath; the MRA of the cerebral vascular is unremarkable (**c**)

**Fig. 4:**
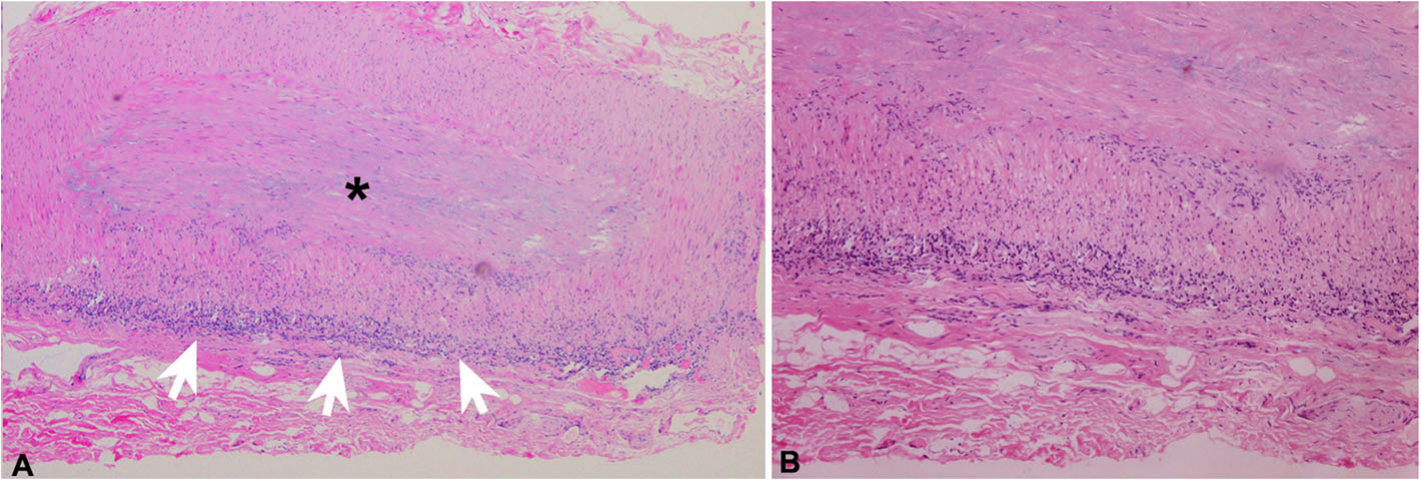
Histopathology of right temporal arterial biopsy showing the almost occlusion of the luminal (asterisk), and diffused infiltration of inflammation by lymphocytes, macrophages and multinuclear giant cells in the arterial wall (white arrows). (**a**): H&E × 40. (**b**): H&E × 100

The routine laboratory tests showed slight decreased RBC (3.09 × 10^12^/L, normal: 4–5.5) and Hb (92 g/l, normal:120–160),with normal platelet count (170 × 10^9^/L, normal:100–300);dramatic elevated erythrocyte sedimentation ratio (ESR: 100 mm/h, normal: 0–15) and C-reactive protein (CRP: 39.27 mg/L, normal: 0–5). The ocular blood flow detected by color duplex ultrasonography (CDUS) revealed decrease of posterior ciliary artery, central retinal artery, ophthalmic artery in both eyes. CDUS of the bilateral superficial temporal arteries with high-frequency linear probe revealed inflammation of the vessel wall as a hypo-echoic concentric ring, which referred as the “halo sign” [8] (Fig. 2). The orbital fat-suppression T1-weighted magnetic resonance imaging (MRI) with contract only showed the enhancement of the optic nerve sheath in the left eye, without inflammatory and mass occupation lesions. The MRA were also unremarkable (Fig. 3). The right temporal artery biopsy was performed and revealed the occlusion of the luminal owing to the intimal proliferation and infiltration (Fig. 4). Methylprednisolone 1 g/d was intravenous for 3 days followed by prednisone 1 mg/kg/d. The vision acuity maintained NLP bilateral after treatment, whereas the headache and jaw pain disappeared. The oral prednisone was weaned and methotrexate was added as the immunosuppressive agent for long treatment. The patient was followed more than one year and there was no other new systematic symptoms, but with the permanent blindness eventually.

## Discussion and conclusion

GCA represents a rare systematic granulomatous vasculitis in Asian, with fever and headache as the most prominent symptoms at onset [5, 6]. An arteritic anterior ischemic optic neuropathy (A-AION) from inflammatory occlusion of posterior ciliary artery is the most common cause of permanent visual loss. Attaseth et al. [9] had reported six A-AION patients in Thailand with biopsy-proven GCA and Cha et al. reported one case from Koreans [10]. To our knowledge, this is the first report of A-AION due to GCA confirmed with biopsy in Chinese patient.

The clinical features and visual outcome of our Chinese patient is not different from those described in Caucasian or Asians from Thailand and Korea [11]. The rapid progressed visual loss in elderly with headache, the “chalky white” pallid disc, as well as the evaluated ESR and CRP, the presumed GCA is not very difficult for ophthalmologists to made, even though patients without the systemic manifestation of GCA.

According to the criteria for diagnose of GCA in 1990, the biopsy is not the necessary condition, but it’s still the gold standard for GCA [12]. Because of the extremely low prevalence of GCA in Asian, the other types of vasculitis such as Wegener granulomatosis, nodular polyarteritis, or Behcet disease should be as the differentiations, and polymyalgia rheumatic as an overlap with GCA. Murchison et al. suggested that all patients suspected of having GCA should undergo a temporal artery biopsy [13]. Some authors commented on the “rare” of GCA in Asian populations is likely to be under-recognized and misdiagnoses, owing to the low biopsy rate [14]. Therefor the CDUS as an noninvasive vascular imaging has an important role in the diagnostic and work-up of large-vessel vasculitis, especially in GCA patients. We also utilized the ultrasound for evaluating the superficial temporal arteries before the biopsy was performed. Using the high-frequency linear probe, the inflammation of the vessel wall can be detected as a hypo-echo concentric ring, which referred as “halo sign”. More clinical evaluation and compared study will be help to obtain a normal temporal arteries normal value in Asian patients.

Although the MRI of the optic nerve would not help to differentiate the arteritic AION from the N-AION, the perio-optic nerve sheath enhancement was also observed in our patient, just as reported in Asian patients before [9]. The unremarkable diffused weighted imaging of the optic nerve of the orbit part can help ophthalmologists to exclude the posterior optic neuropathies due to other inflammatory or infiltration [15].

Because GCA is a rapid progressive irreversible blindness condition, although very rare in Asian patients, the vigilant for quick ESR and CRP tests is very important. The high dose of corticosteroid treatment should not be delayed while waiting for the result of biopsy.

In conclusion, although A-AION due to GCA is very rare in Asian patients, the sudden devastating visual loss with headache in elderly, the white chalk pallid disc, and the evaluated ESR and CRP will differentiate it from the common N-AION type. We emphasize that noninvasive ultrasound and MRI will be a very promising methods for work-up GCA in Asian in case the biopsy is impossible.

## Abbreviations

A-AION: Arteritic ischemic optic neuropathy
AION: Anterior ischemic optic neuropathy
CDUS: Color duplex ultrasonography
CRP: C-reactive protein
ESR: erythrocyte sedimentation rate
GCA: Giant cell arteritis
MRI: Magnetic resonance imaging
N-AION: nonarteritic ischemic optic neuropathy
